# Genome-wide identification of the rubber tree superoxide dismutase (*SOD*) gene family and analysis of its expression under abiotic stress

**DOI:** 10.7717/peerj.14251

**Published:** 2022-10-24

**Authors:** Wencai Yu, Guanghong Kong, Jinquan Chao, Tuo Yin, Hai Tian, Huajin Ya, Ligang He, Hanyao Zhang

**Affiliations:** 1 Key Laboratory for Forest Resources Conservation and Utilization in the Southwest Mountains of China, Ministry of Education, Southwest Forestry University, Kunming, Yunnan Province, China; 2 Yunnan Institute of Tropical Crops, Jinghong, Yunnan Province, China; 3 Ministry of Agriculture and Rural Affairs Key Laboratory of Biology and Genetic Resources of Rubber Tree, Chinese Academy of Tropical Agricultural Sciences, Haikou, Hainan Province, China

**Keywords:** *Hevea brasiliensis*, Superoxide dismutase (SOD), Abiotic stress, ROS, Gene expression, Gene structure, Cis-elements, Gene ontology

## Abstract

**Background:**

The rubber tree (*Hevea brasiliensis*) is the only species capable of producing high-quality natural rubber for commercial use, and is often subjected to various abiotic stresses in non-traditional rubber plantation areas. Superoxide dismutase (SOD) is a vital metalloenzyme translated by a *SOD* gene family member and acts as a first-line of protection in plant cells by catalysing the disproportionation of reactive oxygen species (ROS) to produce H_2_O_2_ and O_2_. However, the *SOD* gene family is not reported in rubber trees.

**Methods:**

Here, we used hidden markov model (HMM) and BLASTP methods to identify SOD genes in the *H. brasiliensis* genome. Phylogenetic tree, conserved motifs, gene structures, cis elements, and gene ontology annotation (GO) analyses were performed using MEGA 6.0, MEME, TBtools, PlantCARE, and eggNOG database, respectively. *HbSOD* gene expression profiles were analysed using quantitative reverse transcription polymerase chain reaction (qRT-PCR).

**Results:**

We identified nine *HbSOD* genes in the rubber tree genome, including five *HbCSDs*, two *HbFSDs*, and two *HbMSDs*. Phylogenetic relationship analysis classified the SOD proteins from the rubber tree and other related species into three subfamilies. The results of gene structure and conserved motif analysis illustrated that most *HbSOD* genes have similar exon-intron numbers and conserved motifs in the same evolutionary branch. Five hormone-related, four stress-related, and light-responsive elements were detected in the *HbSODs’* promoters. *HbSODs* were expressed in different tissues, gradually increased with leaf development, and were abundantly expressed in mature leaves. *HbCSD2* and *HbCSD4* was significantly upregulated under low and high temperatures, and salt stress, except for *HbCSD2*, by heat. Furthermore, most *HbSOD* genes were significantly upregulated by drought, except *HbMSD2*. These findings imply that these genes may play vital roles in rubber tree stress resistance. Our results provide a basis for further studies on the functions of *HbSOD* genes in rubber trees and stress response mechanisms.

## Introduction

Plants grow in constantly changing environments and are exposed to various abiotic stresses during growth, development, and production, such as extreme temperature, drought, waterlogging, salt, and heavy metal poisoning (Zhu, 2016). Under normal environmental conditions, the levels of reactive oxygen species (ROS) level are low and in dynamic equilibrium in plant cells, although under abiotic stresses, the dynamic equilibrium of ROS becomes disrupted and a large amount of ROS will be accumulated (Hasanuzzaman et al., 2020; Nadarajah, 2020). ROS include hydrogen peroxide (H_2_O_2_), superoxide anions (·O_2_^−^), hydroperoxyl radicals (·HO_2_^−^), hydroxyl radicals (·OH^−^), and alkoxy radical (Del Rio, 2015; Waszczak, Carmody & Kangasjarvi, 2018). Enrichment of ROS can cause protein oxidation, DNA damage, lipid peroxidation, and even cell death (Choudhury et al., 2017; Gill et al., 2015; Gill & Tuteja, 2010).

To adapt to adverse environmental conditions, plants have evolved complex and efficient antioxidant defences, including enzymatic and non-enzymatic systems (Rajput et al., 2021). The enzymatic system is a vital component and consists of many enzymes, such as SOD, CAT and POX (Gill & Tuteja, 2010; Pereira, 2016; Rajput et al., 2021). SOD acts as the first line of protection in plant cells and can catalyse superoxide anion (·O_2_^−^) discordance to generate O_2_ and H_2_O_2_, effectively clean up ROS and protect cells from harm, and is an antioxidant enzyme for scavenging ROS in plant cells (Rajput et al., 2021). SOD is a vital metalloenzyme and is categorized into three main subgroups according to different metal cofactors (Cu, Zn, Mn, and Fe), namely Cu/Zn-SOD, Fe-SOD, and Mn-SOD (Fink & Scandalios, 2002). SODs localized in various cellular organs. For instance, Cu/Zn-SOD subfamily members are localized in the cytoplasm, chloroplasts, peroxisomes, and extracellular regions. whereas Fe-SODs are localized in chloroplasts, and Mn-SODs are localized in the mitochondria and peroxisomes (Huseynova, Aliyeva & Aliyev, 2014; Rajput et al., 2021; Stephenie et al., 2020).

Plant *SOD* genes can be induced by abiotic stresses to cope with adverse environments (Choudhury et al., 2017; Gill et al., 2015; Gill & Tuteja, 2010; Rajput et al., 2021; Wang et al., 2016b; Waszczak, Carmody & Kangasjarvi, 2018). For example, in *Arabidopsis thaliana*, *SOD* genes increased significantly under oxidative stress (Kliebenstein, Monde & Last, 1998). Under abiotic stresses (salinity, cold, waterlogging, and drought), eight *BnSOD* genes are significantly up-regulated in rapeseed (Su et al., 2021). Most *SOD* genes exhibit transcriptional responses to drought stress in poplar tree (Molina-Rueda, Tsai & Kirby, 2013). In tea plants, most *SOD* genes are induced after exposure to cold and drought stress (Zhou et al., 2019). In addition, according to Shiraya et al. (2015), in rice, *OsMSD1* overexpression enhanced tolerance to heat, and in contrast, *MSD1*-knockdown rice was notably susceptible to heat stress. *TaSOD2* overexpression in both wheat and *Arabidopsis* increased SOD activity and enhanced resistance to salt and oxidative stress (Wang et al., 2016a). After heterologous expression of the *Cu/Zn-SOD* gene from *Jatropha curcas*, salt tolerance in transgenic *A. thaliana* was improved (Liu et al., 2015). Moreover, the joint overexpression *MeCu/ZnSOD* and *MeCAT1* improved cold and drought tolerance in cassava (Xu et al., 2013). In a recent study, Han et al. (2019) over-expressed *LkSOD2*, *LkSOD4*, and *LkSOD6* genes from the Japanese larch in *A. thaliana* and improved the resistance to salt stress. Taken together, these studies show that *SOD* genes play vital roles in the plant responses to abiotic stresses. Recently, with the rapid development of genome sequencing, the *SOD* gene family members of different species were comprehensively identified at the genome-wide level in many plant species, *e.g., Brassica napus* (Su et al., 2021), *A. thaliana* (Kliebenstein, Monde & Last, 1998), *Brassica rapa* (Iqbal Qureshi et al., 2021), *Salvia miltiorrhiza* (Han et al., 2020), *Dendrobium catenatum* (Huang et al., 2020), *Camellia sinensis* (Zhou et al., 2019), *Zostera marina* (Zang et al., 2020), *Vitis vinifera* (Hu et al., 2019), *Gossypium hirsutum* (Wang et al., 2017) and *Solanum lycopersicum* (Feng et al., 2016).

Natural rubber is a vital and renewable raw industrial material (Yamashita & Takahashi, 2020). To date, at least 2,500 species of plants can synthesise natural rubber (van Beilen & Poirier, 2007), but only the rubber tree (*Hevea brasiliensis*) is capable of producing high-quality commercial natural rubber and constitutes more than 98% of the global rubber production (Supriya & Priyadarshan, 2019). However, rubber trees are frequently subjected to numerous abiotic stresses that affect the plant growth and development, ultimately reducing the latex yield (Mohamed Sathik et al., 2018). To date, there are no reports regarding the identification of the *SOD* gene family in rubber tree. Therefore, in this study, a genome-wide comprehensive analysis of *SOD* genes in the rubber tree genome was performed and physicochemical properties and bioinformatics were analysed. Furthermore, tissue-specific expression and expression patterns under various abiotic stresses are comprehensively analysed. This study lays the foundation for further insight into the biological functions of the *SOD* gene family members and molecular breeding for stress resistance.

## Materials and Methods

### Identification of SOD gene family in rubber tree

In this work, we identified the *SOD* gene family in the rubber tree genome in two ways. Rubber tree genome sequences were downloaded from the NCBI database (Tang et al., 2016). In addition, HMM files of Cu/ZnSOD (PF00080) and Fe/MnSOD (PF00081, PF02777) were obtained from the Pfam database (El-Gebali et al., 2019). Rubber tree protein database was scanned using the HMMER software (version 3.2.0) (http://hmmer.org/download.html) (Potter et al., 2018) with the SOD HMM files as reference sequences to search for predicted HbSOD proteins. Second, the protein sequences of the *A. thaliana SOD* family members were downloaded from the *Arabidopsis* genome database (TAIR) (Rhee et al., 2003). Subsequently, these sequences were used as seed sequences to search the rubber tree protein database for candidate *HbSOD* using BLASTP with an e-value of 1e^−5^. For further identification, these obtained sequences were verified in their conserved structural domain by Pfam, SMART (Letunic, Khedkar & Bork, 2021), and CDD (https://www.ncbi.nlm.nih.gov/Structure/cdd/). Finally, all candidate *HbSOD* genes were combined based on the HMMER and BLASTP results and incomplete domains and overlapping domains were removed. The physical and chemical properties of HbSOD proteins were analysed using the online tool ExPASY ProtParam (https://web.expasy.org/protparam). Subcellular localisation of *HbSOD* genes were predicted using ProtComp 9.0 (http://linux1.softberry.com/).

### Gene structures, conserved motifs, cis-elements and GO annotation analysis

The MEME (Bailey et al., 2006) online tool was used to predict the protein-conserved motif, with the motif value set to eight and other parameters at default, and then the conserved motifs and gene structures were mapped using TBtools software (Chen et al., 2020). Additionally, according to the previous studies (Su et al., 2021; Zang et al., 2020), we extracted a 2,000 bp sequence upstream of the *HbSOD* genes from the start codon from the *H. brasiliensis* genome database using TBtools as the promoter segment, and the cis elements were predicted using the online analysis tool PlantCARE (Lescot et al., 2002). To analyse the functional annotation of the *HbSOD* genes, all HbSOD protein sequences were uploaded to the eggNOG database (Cantalapiedra et al., 2021) for Gene Ontology (GO) function annotation, and then GO enrichment analysis was performed using TBtools.

### Phylogenetic analysis

The evolutionary relationship of the *HbSOD* gene family was analysed, by downloading the well-known SOD protein sequences from the NCBI database of *Ricinus communis*, which belongs to the same family (Euphorbiaceae) as the rubber tree, and *Populus euphratica*, which is a species adaptable to harsh environmental conditions in woody plants (Zhang et al., 2021). A phylogenetic tree with *H. brasiliensis* and *A. thaliana* SOD protein sequences was then constructed. Sequences were aligned using the ClustalW program of the MEGA 6.0, and the phylogenetic tree was constructed using the neighbor-joining (NJ) method with a bootstrap value of 1,000 and the other parameters as default (Kumar et al., 2018). Then, the phylogenetic tree was prettified by Evolview v3 (Subramanian et al., 2019).

### Plant materials and stress conditions

In this study, asexual seedlings of a large-scale cultivar ‘GT1’ of rubber tree, planted at the experimental base of the Yunnan Institute of Tropical Crop Science (Jinghong, China), were used as experimental material. Abiotic stress treatments were performed when the plantlets had developed one extension unit and the leaves were mature in nutritious bags. These plantlets were then transferred to a growth chamber under the conditions of 16 h light/8 h dark cycle, 28 °C, and 80% humidity. After 48 h, the plants were divided into five groups, each group containing 105 plantlets, that were exposed to different treatment conditions. According to our recent study (Li et al., 2019), two groups of seedlings were transferred to 4 °C and 40 °C growth chambers for low- and high temperature treatments, respectively. One group of plantlets was removed from the nutritious bags and cultivatable soil, and bare roots were placed in flowerpots for drought stress treatment. We prepared a 300 mM NaCl solution and poured 300 mL into each plant pot for salt stress treatment. One group without stress treatment (28 °C, 80% humidity), served as the control. The experiment was performed with three biological replicates of five seedlings each. Leaves were collected from the control and treated seedlings at 0, 1, 3, 6, 12, 24, and 48 h after stress treatment. All the treatments were performed with three biological controls and replicates. Samples were quickly placed in liquid nitrogen and stored at −80 °C for storage. To analyse expression profiles of *HbSOD* genes in different tissues, we selected 10-year-old plants of the rubber tree variety ‘GT1’ and selected budburst, copper-drown, light-green, mature leaves, barks, and roots.

### RNA extraction and qRT-PCR analysis

Total RNA was extracted from each sample using the RNAprep Pure Plant Plus Kit (DP441; Tiangen, Beijing, China). RNA was measured using a NanoDrop2000 Spectrometer (Thermo Fisher Scientific, Waltham, MA, USA), and RNA integrity was evaluated by 1.0% agarose gel electrophoresis. RevertAid TM First Strand cDNA Synthesis Kit (K1622; Thermo Fisher Scientific, Waltham, MA, USA) was used to reverse transcribe RNA (2 μg per reaction) into complementary DNA (cDNA), which was then diluted 10 times for quantitative reverse transcription polymerase chain reaction (qRT-PCR) analysis. The specific primers for the *HbSOD* genes were designed using Premier 5.0 software and delivered to Sangon Biotech (Shanghai, China) for synthesis (Table S1). qRT-PCR analyses were performed on a qTOWER 2.2 real-time fluorescence quantitative system (Analytik Jena, Jena, Germany). *Actin7a* gene of rubber tree was used as an internal control. Three technical replicates were performed for each cDNA sample. The cycle threshold (CT) data obtained from qRT-PCR were computed using the 2^−ΔΔCT^ method (Liu et al., 2018) to determine the relative expression of genes. The heat maps were constructed using the TBtools software.

## Results

### Identification of the *SOD* gene family in rubber tree

In this study, nine *HbSOD* genes were identified from the rubber tree genome, including five *HbCSDs*, two *HbFSDs* and two *HbMSDs*, which were named *HbCSD1, HbCSD2, HbCSD3, HbCSD4*, *HbCSD5*, *HbFSD1*, *HbFSD2*, *HbMSD1*, and *HbMSD2* (Table 1; Table S2). The results of physicochemical property analysis showed that the CDS of *HbSODs* ranged from 459 bp (*HbCSD1, HbCSD2*) to 975 bp (*HbCSD5*). The full-length of protein sequences ranged from 152 aa (*HbCSD1*) to 324 aa (*HbCSD5*). The molecular weight of *HbSODs* ranged from 15.26 kDa (*HbCSD1*) to 34.42 kDa (*HbCSD5*) and the isoelectric points ranged from 5.23 (*HbFSD1*) to 9.56 (*HbCSD4*) (Table 1). The predicted subcellular localization results revealed that *HbCSD1-HbCSD5* were localised in the cytoplasm, *HbFSD1* and *HbFSD2* in chloroplasts, and *HbMSD1* and *HbMSD2* in mitochondria (Table 1).

**Table 1:** The detailed information of *SOD* genes identified in the *Hevea brasiliensis* genome. *HbCSD4* (scaffold0046_2534972) was identified in the local rubber tree genome database of the Chinese Academy of Tropical Agricultural Sciences (CATAS) and its sequence has been uploaded to GenBank: ON011077.

Gene name	Gene ID	Accession number	ORF length (bp)	Protein physicochemical characteristics			Predicted Pfam domain	Subcellular prediction by PC
				Length (aa)	Mw (kDa)	Pl		
*HbCSD1*	scaffold0638_497321	XM_021782136.1	459	152	15.26	5.60	CZ	Cytoplasm
*HbCSD2*	scaffold0117_29717	XM_021813881.1	477	158	16.32	5.72	CZ	Cytoplasm
*HbCSD3*	scaffold0549_93226	XM_021836413.1	822	273	28.59	9.56	CZ	Cytoplasm
*HbCSD4*	scaffold0046_2534972	ON011077	669	223	24.05	7.56	HMA, CZ	Cytoplasm
*HbCSD5*	scaffold4109_2853	XM_021808273.1	975	324	34.42	6.85	HMA, CZ	Cytoplasm
*HbFSD1*	scaffold0099_680412	XM_021812278.1	915	304	34.23	5.23	IMA, IMC	Chloroplast
*HbFSD2*	scaffold0329_803149	XM_021826019.1	876	291	34.36	7.58	IMA, IMC	Chloroplast
*HbMSD1*	scaffold0387_121763	XM_021829101.1	702	233	25.84	7.10	IMA, IMC	Mitochondrion
*HbMSD2*	scaffold0427_434785	XM_021831459.1	708	235	26.11	7.83	IMA, IMC	Mitochondrion

### Gene structures and conserved motifs

**Figure 1:**
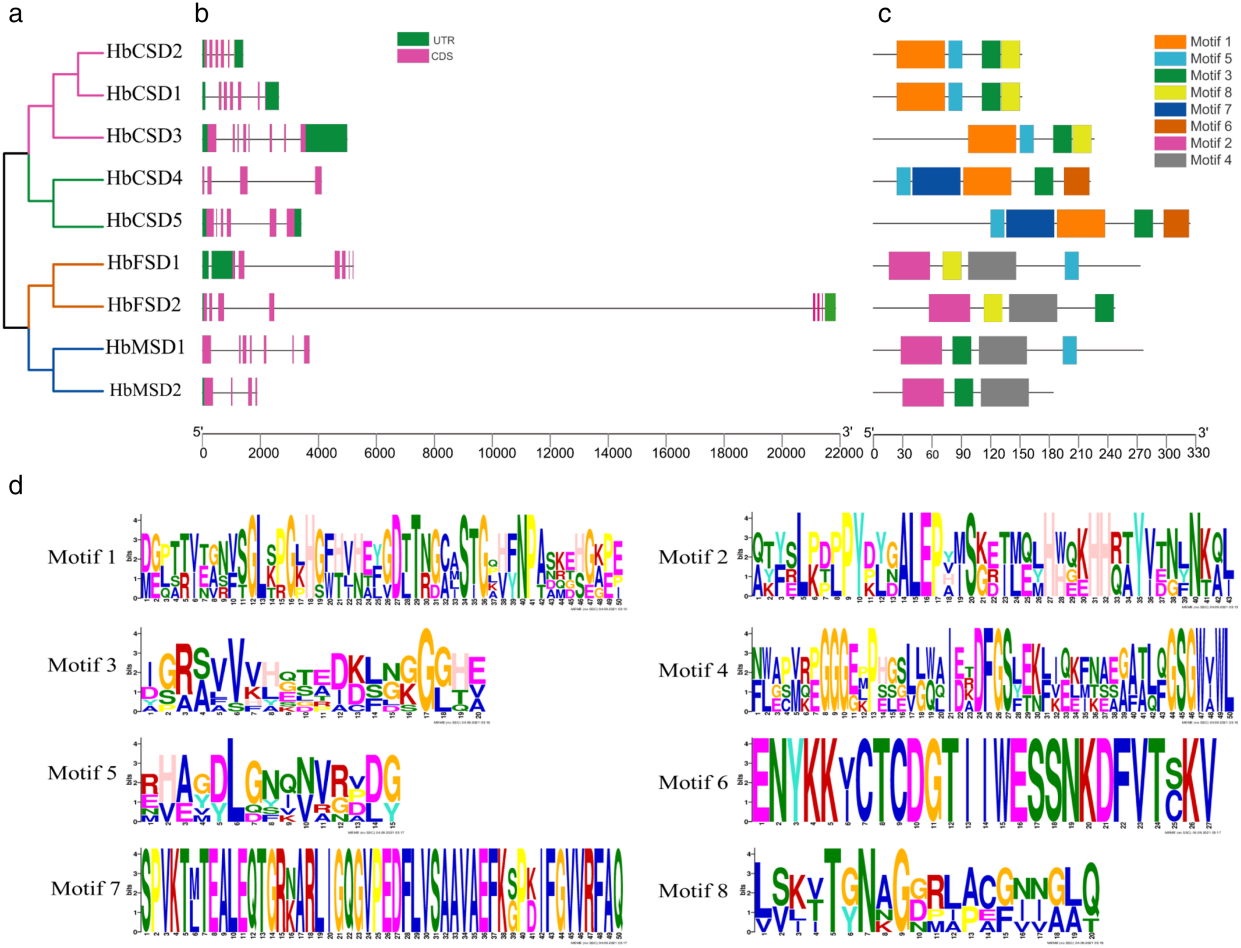
Phylogenetic tree, conserved motifs, and motif logos of HbSODs. (A) Phylogenetic relationships and domain identification. (B) Gene structures of HbSODs. (C) Conserved motif compositions identified in HbSODs. (D) Conserved motif logos in HbSODs.

The study of gene exon/intron patterns assists understanding the evolution of the development of rubber tree SOD family genes. Therefore, we comprehensively analysed the gene structure and conserved motifs of *HbSOD* gene*s*. The results displayed that the number of exons and introns of *HbSOD* genes ranged from four to eight and three to seven, respectively (Fig. 1B). The Cu/Zn-SOD group contained six exons and five introns, except *HbCSD3*, which contained seven introns and eight exons, and *HbCSD4* which contained four exons and three introns. The Fe-SOD subfamily had six to seven exons and five to six introns. The *HbMSD1* contained seven exons and six introns, and the number of exons/introns of the *HbMSD2* gene contained three introns and four exons. In summary, the number of exons and introns of *HbSOD* genes was significantly different but had similar intron/exon patterns in the same evolutionary branch. To further investigate the gene structure and function of *HbSOD* genes, we calculated the motifs of nine HbSOD proteins using MEME software and visualized them using the TBtools software (Fig. 1C). The results showed that eight motifs were identified in *HbSODs* (Fig. 1D; Table S3). Motif 3 was widely distributed across almost all *HbSOD* genes, except for *HbFSD1*. Motif 5 was presented in most proteins of *HbSOD* gene family, except for *HbFSD2* and *HbMSD2*. This indicates that Motif 3 and 5 were relatively conserved in the evolution of HbSOD proteins in the rubber tree. Motif 1 is a unique conserved domain of Cu/Zn-SOD subfamily members, and motif 6 only exists in *HbCSD4* and *HbCSD5*. Members of the Fe-SOD and Mn-SOD subfamilies contained motifs 2 and 4.

### Phylogenetic relationship analysis of *HbSOD* genes

**Figure 2:**
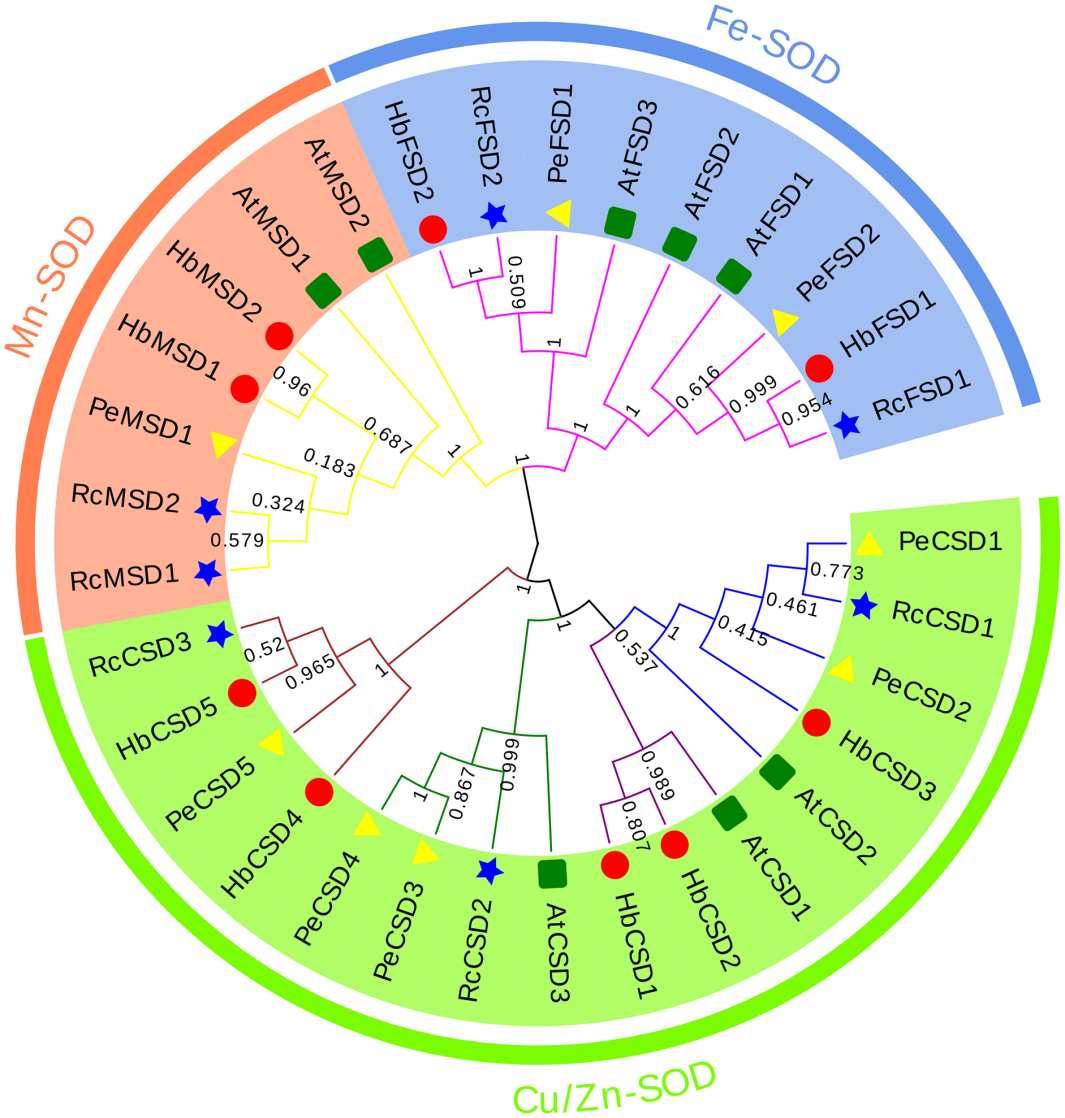
Phylogenetic analysis of SOD proteins from *H. brasiliensis*, *R.communis*, *P. euphratica*, and *A. thaliana*.

A phylogenetic relationship of 32 SOD proteins from *H. brasiliensis* (nine), *A. thaliana* (eight), *R. communis* (seven), and *P. euphratica* (eight) (Table S2) *w*as constructed using the NJ method of MEGA6.0 software. As we can see from the phylogenetic tree (Fig. 2), *SODs* were clustered into three major groups: Cu/Zn-SOD, Fe-SOD, and Mn-SOD. The Cu/Zn-SOD group contained 16 SOD members (three *AtSODs*, five *HbSODs*, five *PeSODs* and three *RcSODs*), Fe-SOD nine members (three *AtSODs*, two *HbSODs*, two *PeSODs* and two *RcSODs*), and Mn-SOD seven members (two *AtSODs*, two *HbSODs*, one *PeSODs* and two *RcSODs*). Further analysis revealed that the Cu/Zn-SOD subfamily was divided into four branches, where *HbCSD4*, *HbCSD5*, *RcCSD3*, and *PeCSD5* were clustered together individually. *HbSODs* were more closely related to *RcSODs* and *PeSODs* in each subclade, particularly in the Fe-SOD and Mn-SOD subfamilies.

### Prediction of cis-elements in promoters of *HbSOD* genes

**Figure 3:**
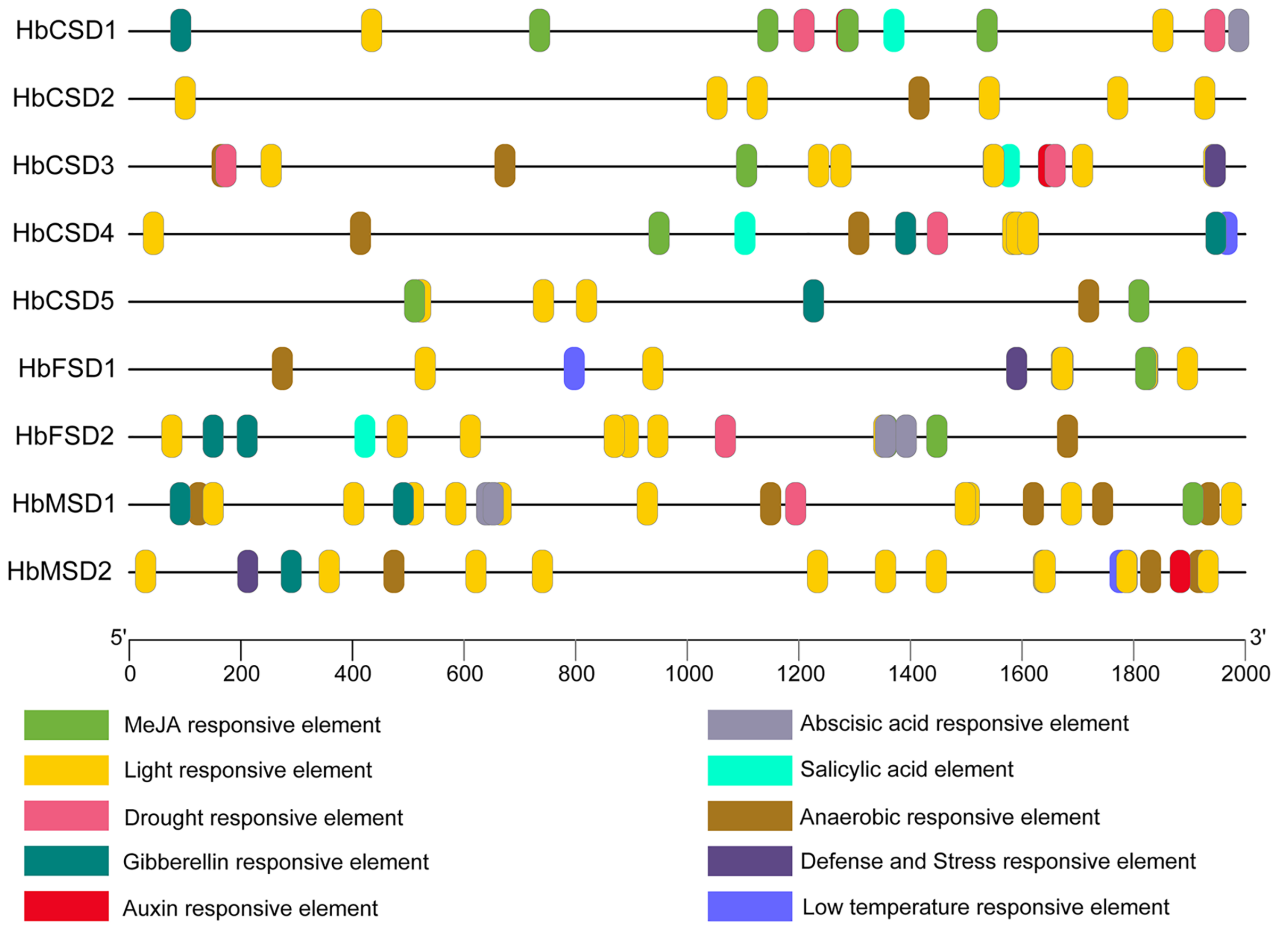
Cis-elements in promoters of the *HbSOD* genes. Different color boxes show different cis-elements related to various hormones and stress-responsive elements.

The analysis of cis elements in the promoter region of *HbSODs* can assist exploration of the potential mechanisms of genes in response to multiple stresses. We predicted the cis elements of the 2,000 bp upstream sequences of the nine *HbSOD* genes, and the details of the cis-acting elements are shown in Table S4. One hundred and fifty-eight cis elements were identified from all *HbSOD* promoters, which were related to hormones and stress responses (Fig. 3; Table S4). Five types of cis-acting elements are related to plant hormone responses to abscisic acid (ABA), auxin, gibberellin (GA), methyl jasmonate (MeJA), and salicylic acid (SA) (Fig. 3). Further analysis showed that most *HbSOD* genes contained MeJA-responsive elements CGTCA-motif and TGACG-motif (except for *HbCSD2* and *HbMSD2*) and ABA-responsive element ABRE (except for *HbCSD2* and *HbCSD5*), GA-responsive elements TATC-box, P-box, and GARE-motif (except for *HbCSD2*, *HbCSD3*, and *HbFSD1*). In summary, MeJA-, ABA- and GA-responsive elements are largely distributed in most *HbSOD* genes, suggesting that *HbSOD* genes may be involved in growth and development as well as stress response regulation mediated by the above plant hormones. Moreover, five different stress-related response elements were identified, including drought, low temperature, stress resistance, defence responce, anaerobic induction, and light response elements. Furthermore, most *HbCSD* genes contain drought stress response elements (MBS), except for *HbCSD2*, *HbFSD1*, and *HbMSD2*. Low-temperature stress response element (LTR) was identified in *HbCSD4*, *HbFSD1*, and *HbMSD2*. *HbCSD3*, *HbFSD1*, and *HbMSD2* contain defence and stress response elements (TCA-rich repeats). Nearly all *HbSOD* genes had an anaerobic induction response element, except for *HbCSD1*.

### Functional annotation analysis of *HbSOD* genes

**Figure 4:**
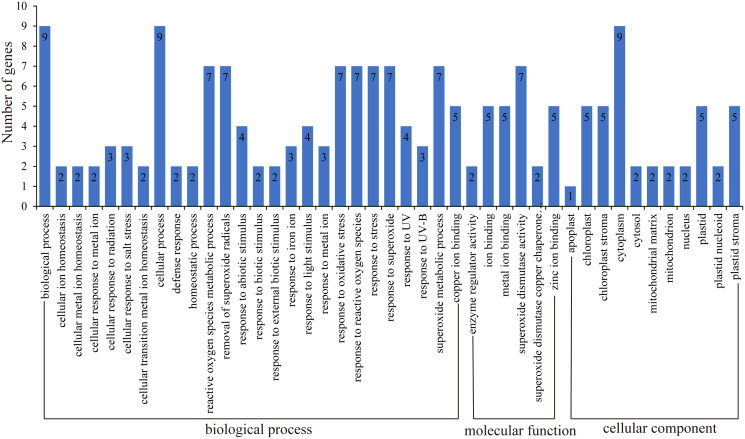
Gene ontology of SOD genes in rubber tree.

GO function annotation of genes allows us to better understand the molecular functions of proteins (Ashburner et al., 2000; Wimalanathan et al., 2018; Wimalanathan & Lawrence-Dill, 2021). Here, we uploaded the HbSOD protein sequences to the eggNOG website for annotation, and TBtools were used for GO enrichment analysis (Fig. 4; Table S5). The results of biological process annotation showed that all nine *HbSODs* were involved in cellular processes (GO:0009987) and biological processes (GO:0008150). Most *HbSODs* were involved in response to superoxide (GO:0000303), response to oxygen radical (GO:0000305), response to stress (GO:0006950), cellular oxidant detoxification (GO:0098869), response to an inorganic substance (GO:0010035), response to oxidative stress (GO:0006979), response to a toxic substance (GO:0009636), response to stimulus (GO:0050896), response to chemicals (GO:0042221), and response to abiotic stimuli (GO:0009628) (Fig.4; Table S5). Molecular function (MF) annotation showed that most *HbSODs* were related to copper ion binding (GO:0005507), superoxide dismutase activity (GO:0004784), zinc ion binding (GO:0008270), oxidoreductase activity (GO:0016491), antioxidant activity (GO:0016209), and metal ion binding (GO:0046872) (Fig. 4; Table S5). Results of cell component annotation showed that *HbSODs* mainly involved cellular components (GO:0005575), cytoplasm (GO:0005737), chloroplast (GO:0009507), and mitochondrion (GO:0005739) (Fig. 4; Table S5). The results of gene function annotation showed that *HbSODs* are enriched not only in the process of responding to various stress and scavenging superoxide radicals but also in cell composition, SOD activity, and metal ion complexes.

### Expression patterns of *HbSOD* genes in various tissues

**Figure 5:**
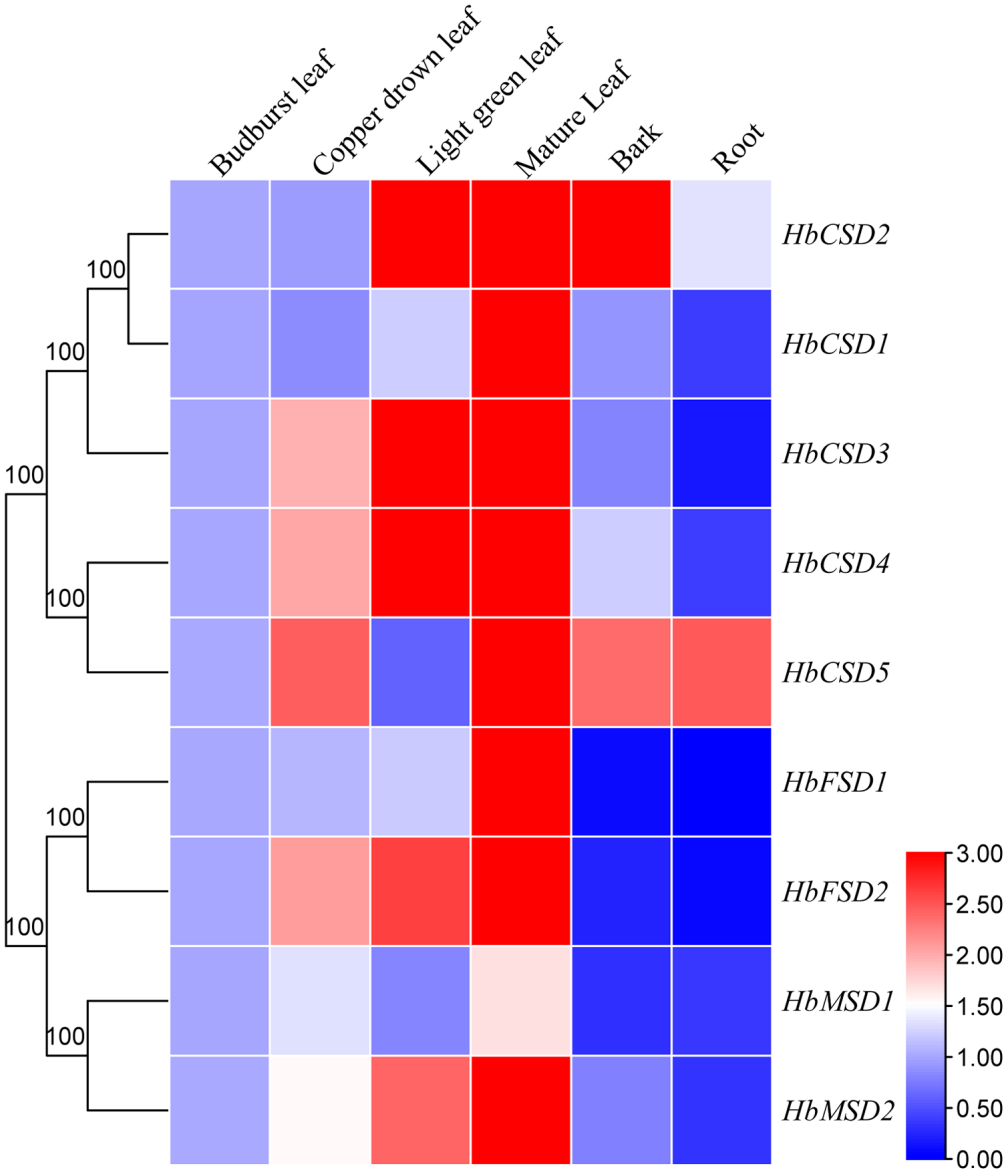
Expression analysis of *HbSOD* genes in several organs. The expression levels were measured with 2^−∆∆CT^ and treated as double normalized using the reference genes and the expression at the bud-burst leaf.

Expression pattern of *HbSOD* genes in different tissues will assist understanding the function of SOD gene family members in rubber trees. We investigated the expression patterns of nine *HbSOD* genes in budburst, copper-drown, light green mature leaves, roots, and bark using qRT-PCR. As shown in Fig. 5, nearly all *HbSOD* genes increased with the development of the leaf and showed higher expression in the mature leaves than in the other developmental stages leaves. We also found that *HbSOD2* and *HbSOD5* were highly expressed in the bark, whereas expression levels of the other *HbSODs* were low. Most *HbSOD* genes had low expression in the roots, whereas *HbSOD5* was high.

### Expression profiles of *HbSOD* genes under abiotic stress

**Figure 6:**
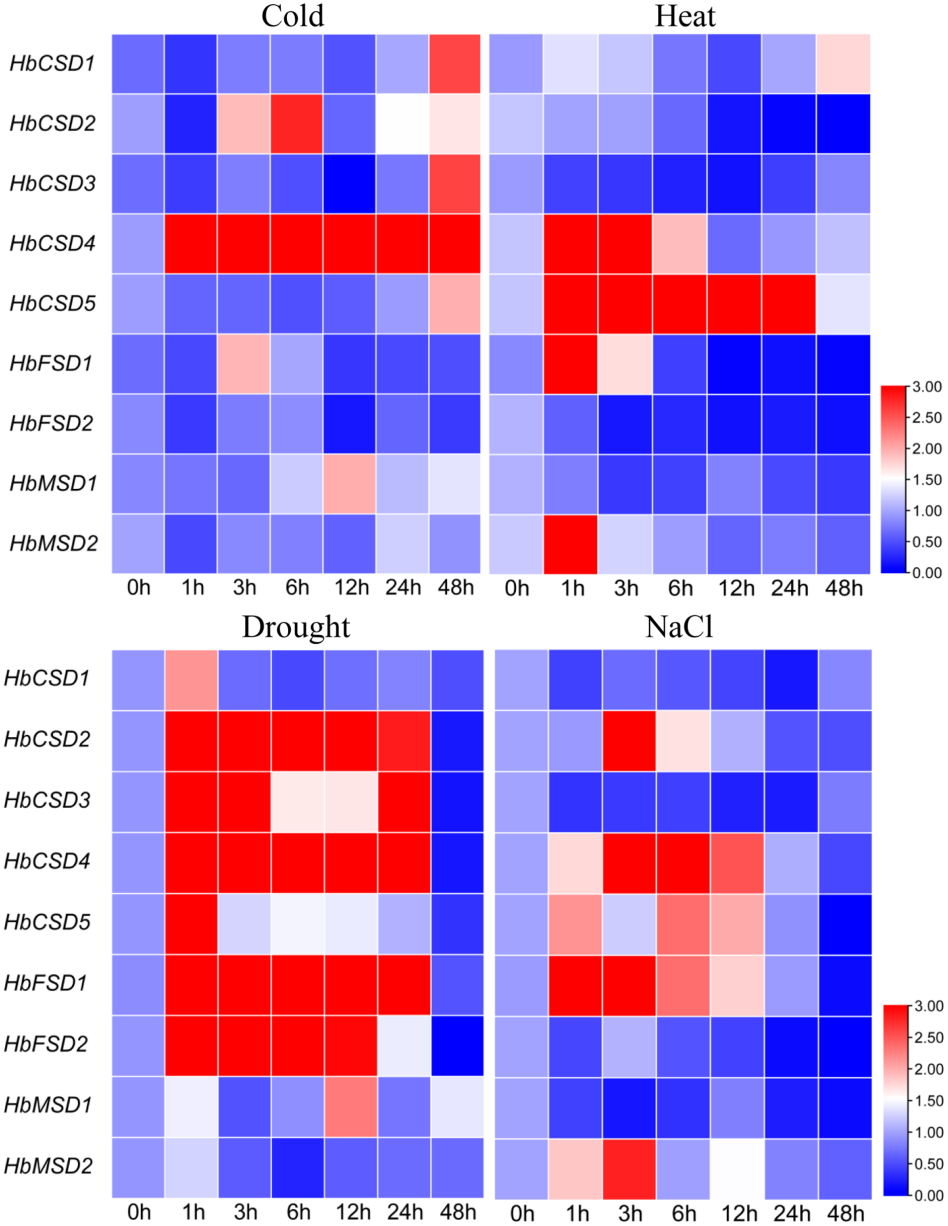
qRT-PCR analysis of the expression patterns of *HbSOD* genes in the leaves under different abiotic stress conditions.

Climate change and the associated adverse abiotic stress conditions (extreme temperature, drought, and salinity) influence rubber tree growth and development, ultimately decreasing rubber yield. To investigate the response of *HbSOD* genes to abiotic stress, we used qRT-PCR to analyse the expression patterns of *HbSODs* under low temperature (4 °C), salt (300 mmol/L NaCl), heat (42 °C), and drought stress. Different genes exhibit different expression patterns under various abiotic stresses conditions. Under low-temperature stress, the expression of *HbCSD2* and *HbCSD4* was significantly upregulated at almost all treatment time points, but not *HbCSD2*, at 1 h (Fig. 6; Table S6). *HbCSD2* and *HbCSD4* reached their maximum expression levels at 6 h after cold treatment and were 2.81 and 16.03 times that of the control, respectively. *HbCSD1*, *HbCSD3*, *HbCSD5*, *HbFSD1*, and *HbMSD1* were significantly upregulated at 48, 48, 48, 3, and 12 h, respectively, and there were no significant changes at other time points. *HbCSD5* was strongly induced by heat stress and maintained a high expression level, reaching a peak at 24 h (36.7 times than of the control) (Fig. 6; Table S6). The *HbCSD4* expression at 1 and 3 h was 9.7 and 5.6 times that of the control, respectively, and there was no significant change in other time points. *HbCSD2*, *HbCSD3*, *HbFSD2*, and *HbMSD1* were downregulated under heat stress (Fig. 6; Table S6). Under drought stress, nearly all *HbSOD* genes had relatively high expression levels, except for some genes (Fig. 6; Table S6). For example, *HbCSD2*, *HbCSD3*, *HbCSD4*, and *HbCSD5* expression was significantly up regulated, except at 48 h time points. Among them, *HbCSD2* and *HbCSD4* reached a peak at 3 h and were 14.0 and 7.8 times that of the control, respectively (Fig. 6; Table S6), and *HbCSD3* and *HbCSD5* reached the top at 1 h and 5.8 and 4.7 times that of the control, respectively. Under salt stress, all *HbSOD* genes were significantly induced or suppressed. The expression of five *HbSOD* genes, including *HbCSD2*, *HbCSD4*, *HbCSD5*, *HbFSD1*, and *HbMSD2* showed similar trends, a ‘first increased and then decreased’ trend (Fig. 6; Table S6). *HbCSD1, HbCSD3, HbFSD2*, and *HbMSD1* were significantly downregulated and showed relatively low expression.

## Discussion

The rubber tree is a vital tropical capital crop that produce rubber. Recently, with the steady growth in global natural rubber consumption, rubber tree planting has gradually developed into non-traditional rubber planting. Therefore, rubber trees often suffer from various abiotic stresses that affect their growth and development, ultimately decreasing rubber yield (Carr, 2011; Silpi et al., 2006). *SOD* genes are a vital gene family that encodes SOD proteins, which can scavenge ROS from cells and serve as the first line of defense in the antioxidant defense system (Fink & Scandalios, 2002; Rajput et al., 2021). In the current study, we identified nine *SOD* gene family members in the whole genome of the rubber tree, including five *Cu/Zn-SODs*, two *Fe-SODs*, and two *Mn-SODs* (Table 1). The number of *SOD* gene family members of the rubber tree was mostly similar to that in *A. thaliana* (8), *S. miltiorrhiza* (8), foxtail millet (8) (Wang et al., 2018), tomato (9) (Feng et al., 2016), cucumber (9) (Zhou et al., 2017), tea plants (10), and less than that in wheat (26) (Jiang et al., 2019), upland cotton (18), and rapeseed (31). Gene duplication events can occur in various ways, including fragment duplication, whole-genome duplication, and tandem duplication, which is one of the main molecular mechanisms of genome evolution to produce genes with new functions (Cannon et al., 2004; Moore & Purugganan, 2003). Therefore, differences in the number of *SOD* genes among plant species may be triggered by gene duplications, and the duplication events might play a vital role in *SOD* gene family expansion.

Numerous research results showed that the *SOD* genes of different species were clustered into three subfamilies (Fink & Scandalios, 2002; Rajput et al., 2021; Sheng et al., 2014). We analysed the evolutionary relationship of SOD proteins in *H. brasiliensis, A. thaliana, R. communis*, and *P. euphratica*, which were clustered into three subfamilies (Fig. 2): Cu/Zn-SOD, Fe-SOD, and Mn-SOD. Further analysis revealed that Cu/Zn-SOD proteins could be divided into four branches, with *HbCSD4*, *HbCSD5*, *RcCSD3*, and *PeCSD5* clustered together (Fig. 2). Conserved domain analysis revealed that *HbCSD4* and *HbCSD5* contained a HMA domain adjacent to the SOD-Cu domain (Fig.1; Table 1). Similar results have been reported in other species, such as *SlSOD4* and BjuACSD4/6-8 (Verma, Lakhanpal & Singh, 2019). The structure and number of introns and exons play vital roles in the evolution of gene families (Xu et al., 2012; Xu et al., 2019). Here, the number of exons and introns of *HbSOD* genes was significantly different; however, they had similar intron/exon patterns in the same evolutionary branch. It may be due to differences in *SOD* genes caused by exon/intron gain/loss, exonisation/pseudo-exonisation, or insertion/deletion in response to different environmental stresses during the evolution of rubber trees (Xu et al., 2012). Moreover, we found eight motifs in *HbSODs* (Fig.1; Table S3). Motif 1 is a unique conserved motif of Cu/Zn-SOD subfamily members. Motif 6 existed only in *HbCSD4* and *HbCSD5*, and motifs 2 and 4 were distributed in the members of the Fe-SOD and Mn-SOD subfamilies. The *HbSODs* in each evolutionary branch had similar conserved motifs. In summary, similarities in conserved patterns and gene structures confirmed the inferred evolutionary relationship. Similar results have also been reported for crops such as corn (Liu et al., 2021) and rapeseed (Su et al., 2021).

Gene expression is regulated by cis elements and transcription factors in the promoter region. An analysis of the cis elements of the promoter of *HbSOD* genes proves helpful for further understanding the function of *HbSOD* genes in coping with abiotic stress. Here, the promoters of *HbSOD* genes were analysed using the online analysis software PlantCARE. At least one plant hormone response element was detected in most *HbSOD* genes (Fig. 3; Table S4). Therefore, *HbSODs* may be involved in the regulation of rubber tree growth, development, and stress response mediated by plant hormones. Nearly all *HbSOD*s contain more than one cis element in response to abiotic stresses (drought, low temperature, defence, *etc*.). The prediction results of the cis-acting element suggested that *HbSOD* genes might play a vital role in the abiotic stress response of the rubber tree. These results were further confirmed by GO functional annotation analysis. Moreover, there have been similar reports on many different crops (Jiang et al., 2019; Su et al., 2021).

In the current study, most *HbSOD* genes increased in expression with leaf development and expressed were highly expressed in mature leaves (Fig. 5). Huang et al. (2020) reported that most *DcaSOD* genes were highly expressed in the leaves. Similarly, several genes displayed higher expression in poplars (Molina-Rueda, Tsai & Kirby, 2013). In soybean, *GmCSD1*, *GmCSD2*, *GmCSD3*, and *GmCSD6* are highly expressed in the leaves (Lu et al., 2020). These results suggest that *SODs* may be involved in leaf growth and development. At present, the expression patterns of *SOD* genes under abiotic stress conditions have been widely reported. For instance, under low-temperature stress, The *SmCSD2* of *S. miltiorrhiza* was upregulated during the entire stress treatment; under salt stress, almost all *SmSODs* upregulated their expression except *SmFSD2*; under drought stress, the expression patterns of *SmCSD1*, *SmCSD2*, *SmCSD3*, and *SmMSD* increased first and then decreased (Han et al., 2020). In tea plants, under low-temperature (4 °C) stress, the expression of nine *CsSOD* genes was significantly up-regulated; *CsCSD2*, *CsCSD3*, *CsCSD6*, and *CsCSD7* were upregulated under drought stress, suggesting that *CsSOD*s may be play a vital role in tea plants in response to cold stress and drought stress (Zhou et al., 2019). In soybeans, only *GmFSD2* expression was significantly upregulated in 13 *SOD* genes under low-temperature stress, and *GmFSD3*, *GmFSD5*, and *GmCSD5* were upregulated under salt stress (Lu et al., 2020). In watermelon and melon, most *SOD* genes were upregulated by cold, drought, and salt stress; but different members had different response modes under varying stresses (Zhang, Ding & Wei, 2021). These results suggest that *SODs* play vital roles in plants against the adverse environments and that homologous genes respond differently to stress in different plants. Here, the expression patterns of *HbSOD* genes under four abiotic stresses (cold, heat, drought, and salt) were tested using qRT-PCR. The results showed that nine *HbSOD* genes were differentially expressed under different stresses conditions. For example, under cold stress, most *HbSODs* were upregulated to varying degrees, especially *HbCSD4*, which was significantly upregulated at nearly all treatment time points (Fig. 6; Table S6). and the expression level was 16 times higher than that of the control after 6 h of treatment (Fig. 6; Table S6). Furthermore, the involvement of the low-temperature-responsive cis element LTR was detected in *HbCSD4*, indicating that *HbCSD4* might play a vital role under cold stress. Under heat stress, all genes had different degrees of induced expression (Fig. 6; Table S6), in which *HbCSD4* and *HbCSD5* were strongly induced and up-regulated by heat stress. All genes, except *HbMSD2* differentially increased their expression levels under drought stress (Fig. 6; Table S6), and *HbCSD2*, *HbCSD3*, *HbCSD4*, and *HbFSD1* significantly up-regulated, except at 48 h time points. At least one involved drought stress-responsive cis elements including MBS, ABA-responsive element (ABRE), and defence and stress response were identified in all *HbSOD* genes, which could explain the significantly upregulated expression of *HbCSD2*, *HbCSD3*, *HbCSD4*, and *HbFSD1* under drought stress. The *HbCSD2*, *HbCSD4*, *HbCSD5*, *HbFSD1*, and *HbMSD2* expression was differentially upregulated and showed a ‘up-then-down’ expression trends under salt stress (Fig. 6; Table S6). The *HbCSD2* and *HbCSD4* levels were highly significant in response to multiple stresses. This suggests that *HbCSD2* and *HbCSD4* may be vital genes in the rubber tree in response to various abiotic stresses. Further studies are required to elucidate the full function of *HbSODs*.

## Conclusions

Here, we identified nine *SOD* genes in the whole genome of the rubber tree that could be clustered into three subgroups: Cu/Zn-SOD, Fe-SOD, and Mn-SOD. To gain further insight, we predicted gene structures and conserved motifs, and found similar exon/intron patterns and conserved motifs within the same evolutionary branch. Cis elements and GO annotation analysis suggested that *HbSOD* genes may play a vital role in abiotic stress response of the rubber tree. Additionally, the expression of the majority of *HbSOD* genes increased gradually with leaf development and was highly expressed in mature leaves. *HbCSD2* and *HbCSD4* were significantly upregulated under low-, high-temperature and salt-stress conditions. Most genes expression levels were significantly upregulated under drought stress. The results of our study provide a basis for further studies on the biological functions of *HbSOD* genes in rubber tree growth, development, and responses to abiotic stresses.

## Supplemental Information

10.7717/peerj.14251/supp-1 Supplemental Information 1 The list primer was used for gene expression analysis by qRT-PCR.

10.7717/peerj.14251/supp-2 Supplemental Information 2 The Information of *SOD* family genes in *Arabidopsis thaliana*, *Populus euphratica*, *Ricinus communis*.

10.7717/peerj.14251/supp-3 Supplemental Information 3 Detail information of identified 8 motifs in HbSOD proteins.

10.7717/peerj.14251/supp-4 Supplemental Information 4 Information of hormone- and stress-related cis-elements detected in the promoters regions of *HbSOD* genes.

10.7717/peerj.14251/supp-5 Supplemental Information 5 The GO enrichment analysis of *HbSOD* genes.

10.7717/peerj.14251/supp-6 Supplemental Information 6 The expression profiles of *HbSOD* genes under various stress treatments.

## Additional Information and Declarations

### Data Availability

The following information was supplied regarding data availability: The raw measurements are available in the Supplemental Files.
