# Supplementary material for: Genome-wide identification of the rubber tree superoxide dismutase (SOD) gene family and analysis of its expression under abiotic stress
Source: PeerJ. 2022 Oct 24;10:e14251. doi: 10.7717/peerj.14251 (PMC9610661; doi:10.7717/peerj.14251)
Supplement: Supplemental Information 1 [file peerj-10-14251-s001.docx]

| **Table S1. The list primer was used for gene expression analysis by qRT-PCR.** | | | |
| --- | --- | --- | --- |
| **Gene name** | **Gene ID** | **Forward Primer Sequence (5'-3')** | **Reverse Primer Sequence (5'-3')** |
| *HbCSD1* | LOC110633502 | CCTGGGCTTCATGGATTCC | TGAACTACAACTGCCCTTCCAAT |
| *HbCSD2* | LOC110656904 | ATTACCAGCAGTGAGGGCATTA | AGGGTTGAAATGTAGCCCAGTC |
| *HbCSD3* | LOC110673315 | AATATTAGTCATCACCCAGCGCT | TTGGGTCAAAGTAACAACGCC |
| *HbCSD4* | ON011077 | TTCGCCAGCGTGAAACAGA | GCATATCTTTTTGTAGTTCTCGCC |
| *HbCSD5* | LOC110652652 | TCACCTGGGAAACACGGCT | TGTTCCCAGGTCGCCAAGT |
| *HbFSD1* | LOC110655821 | TGTGGTGAAGAGTTCCAATGCT | TCCTCTGTTCTTCTTTTTCTCTCTCA |
| *HbFSD2* | LOC110665778 | AACCAGGAGGCGGTGAGAT | GGAGTGATGGCATTTGATGTTTTA |
| *HbMSD1* | LOC110668065 | GTGACCCGAAAGAACCTACCC | GGCACCATAGTCGTAAGGGAGAT |
| *HbMSD2* | LOC110669694 | CTCTCCGTTCTCTCGTCGCC | CGCCCTTCTCCATAGCATCA |
| *HbActin7a* | HQ260674.1 | GGCACTTTGGTACTCAAGTC | GAAGCATCCCAATCACTCTC |
