# Supplementary material for: Genome-wide identification of the rubber tree superoxide dismutase (SOD) gene family and analysis of its expression under abiotic stress
Source: PeerJ. 2022 Oct 24;10:e14251. doi: 10.7717/peerj.14251 (PMC9610661; doi:10.7717/peerj.14251)
Supplement: Supplemental Information 3 [file peerj-10-14251-s003.docx]

| **Table S3. Detail information of identified 8 motifs in HbSOD proteins** | |  |  |
| --- | --- | --- | --- |
| **Motif name** | **Motif sequence** | **Width** | **E-value** |
| Motif1 | DGPTTVEANVSGLSPGKHGFHVHEFGDTTNGCASTGKHFNPASKEHGAPE | 50 | 9.2E-50 |
| Motif2 | QTYSLPDPPYDLGALEPYISGETLZLHWQKHHRAYVTNLNKQL | 43 | 2.7E-40 |
| Motif3 | IGRAVVVHETEDKLGGGGHE | 20 | 2.4E-14 |
| Motif4 | FWAPVRPGGGELPHGSLGWAIDKDFGSLEKFIZKFNAEGAALFGSGWVWL | 50 | 1.8E-30 |
| Motif5 | RHAGDLGNQNVRPDG | 15 | 1E-12 |
| Motif6 | ENYKKICTCDGTIIWESSNKDFVTCKV | 27 | 1.3E-10 |
| Motif7 | SPVKTLTEALEQTGRKARLIGQGVPEDFLVSAAVAEFKGPDIFGVVRFAQ | 50 | 2.8E-10 |
| Motif8 | LSKVTGNAGGRLACGIIGLQ | 20 | 1.90E-07 |
